# Supplementary material for: Development of chemokine network inhibitors using combinatorial saturation mutagenesis
Source: Commun Biol. 2025 Apr 3;8:549. doi: 10.1038/s42003-025-07778-6 (PMC11969024; doi:10.1038/s42003-025-07778-6)
Supplement: Supplementary file 4 — Supplementary Data 2 [file 42003_2025_7778_MOESM4_ESM.zip › SupplementaryData3/scripts/xAF3.tar!/./script/analysis/JK/plots/ckckrstericplot.pdf]

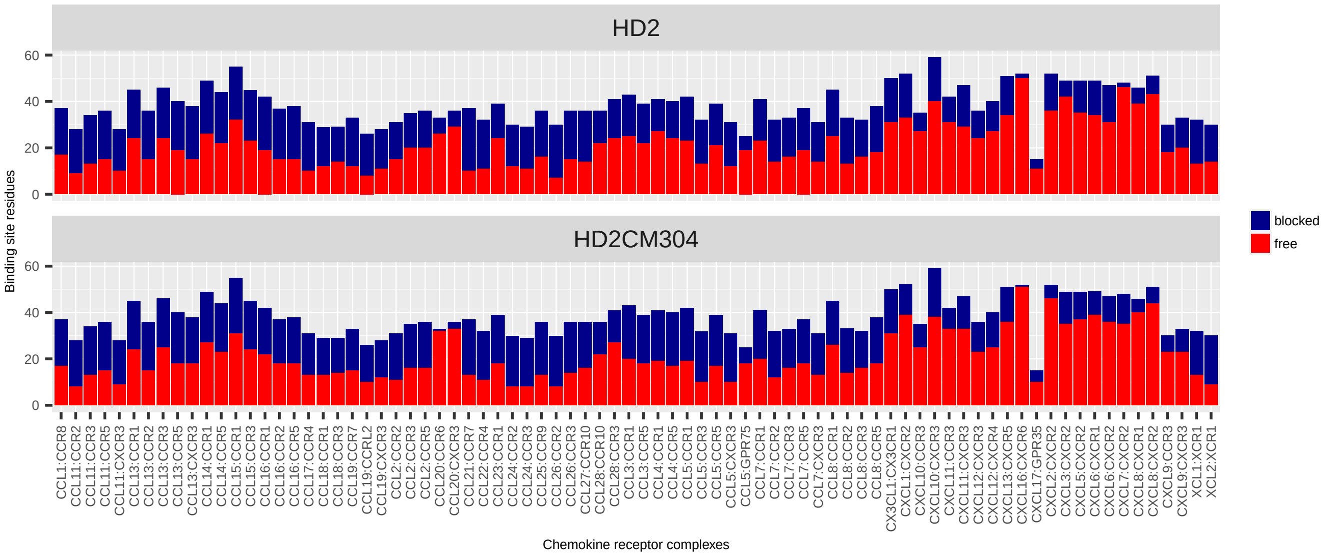
HD2
HD2CM304
Binding site residues
0
20
40
60
blocked
free
Chemokine receptor complexes
CCL1:CCR8
CCL11:CCR2
CCL11:CCR3
CCL11:CCR5
CCL11:CXCR3
CCL13:CCR1
CCL13:CCR2
CCL13:CCR3
CCL13:CCR5
CCL13:CXCR3
CCL14:CCR1
CCL14:CCR5
CCL15:CCR1
CCL15:CCR3
CCL16:CCR1
CCL16:CCR2
CCL16:CCR5
CCL17:CCR4
CCL18:CCR1
CCL18:CCR3
CCL19:CCR7
CCL19:CCRL2
CCL19:CXCR3
CCL2:CCR2
CCL2:CCR3
CCL2:CCR5
CCL20:CCR6
CCL20:CXCR3
CCL21:CCR7
CCL22:CCR4
CCL23:CCR1
CCL24:CCR2
CCL24:CCR3
CCL25:CCR9
CCL26:CCR2
CCL26:CCR3
CCL27:CCR10
CCL28:CCR10
CCL28:CCR3
CCL3:CCR1
CCL3:CCR5
CCL4:CCR1
CCL4:CCR5
CCL5:CCR1
CCL5:CCR3
CCL5:CCR5
CCL5:CXCR3
CCL5:GPR75
CCL7:CCR1
CCL7:CCR2
CCL7:CCR3
CCL7:CCR5
CCL7:CXCR3
CCL8:CCR1
CCL8:CCR2
CCL8:CCR3
CCL8:CCR5
CX3CL1:CX3CR1
CXCL1:CXCR2
CXCL10:CCR3
CXCL10:CXCR3
CXCL11:CCR3
CXCL11:CXCR3
CXCL12:CXCR3
CXCL12:CXCR4
CXCL13:CXCR5
CXCL16:CXCR6
CXCL17:GPR35
CXCL2:CXCR2
CXCL3:CXCR2
CXCL5:CXCR2
CXCL6:CXCR1
CXCL6:CXCR2
CXCL7:CXCR2
CXCL8:CXCR1
CXCL8:CXCR2
CXCL9:CCR3
CXCL9:CXCR3
XCL1:XCR1
XCL2:XCR1
